# Investigation of the Rupture Surface of the Titanium Alloy Using Convolutional Neural Networks

**DOI:** 10.3390/ma11122467

**Published:** 2018-12-05

**Authors:** Ihor Konovalenko, Pavlo Maruschak, Olegas Prentkovskis, Raimundas Junevičius

**Affiliations:** 1 Department of Industrial Automation, Ternopil National Ivan Pul’uj Technical University, Rus’ka str. 56, 46001 Ternopil, Ukraine; icxxan@gmail.com (I.K.); maruschak.tu.edu@gmail.com (P.M.); 2 Department of Mobile Machinery and Railway Transport, Faculty of Transport Engineering, Vilnius Gediminas Technical University, Plytinės g. 27, LT-10105 Vilnius, Lithuania; raimundas.junevicius@vgtu.lt

**Keywords:** image processing, convolutional neural network, dimples of tearing, fracture mechanisms

## Abstract

The research of fractographic images of metals is an important method that allows obtaining valuable information about the physical and mechanical properties of a metallic specimen, determining the causes of its fracture, and developing models for optimizing its properties. One of the main lines of research in this case is studying the characteristics of the dimples of viscous detachment, which are formed on the metal surface in the process of its fracture. This paper proposes a method for detecting dimples of viscous detachment on a fractographic image, which is based on using a convolutional neural network. Compared to classical image processing algorithms, the use of the neural network significantly reduces the number of parameters to be adjusted manually. In addition, when being trained, the neural network can reveal a lot more characteristic features that affect the quality of recognition in a positive way. This makes the method more versatile and accurate. We investigated 17 models of convolutional neural networks with different structures and selected the optimal variant in terms of accuracy and speed. The proposed neural network classifies image pixels into two categories: “dimple” and “edge”. A transition from a probabilistic result at the output of the neural network to an unambiguously clear classification is proposed. The results obtained using the neural network were compared to the results obtained using a previously developed algorithm based on a set of filters. It has been found that the results are very similar (more than 90% similarity), but the neural network reveals the necessary features more accurately than the previous method.

## 1. Introduction

For the non-destructive visual analysis of surfaces, the systems of machine vision are widely used. They are aimed at recognizing visual patterns that are characteristic for defective and undamaged surface fragments. The industrial application of such systems requires reliability, accuracy and speed of response. The methods for detecting defects are classified into four main categories: statistical [1], structural, filter-based [2], and model-based. The techniques based on the analysis of histograms [3,4], co-occurrence matrices [5], and local binary patterns [6,7] are also used. The approaches based on the use of neural networks are used increasingly more often. The latest advances in the field of convolutional neural networks and use of the graphics processors to perform parallel calculations ensure the practical use of such approaches in real production conditions.

Weimer et al. [8] developed a method to identify common features of the image surface, implemented with a two-layer neural network. Features of image are generated by combining multi-resolution analysis and grayscale patch statistics. The detection scheme consists of two major sections. In the training mode, the algorithm uses a dataset of labeled training examples with and without defect regions and extracts random patches from defective and non-defective regions. In the last step, the neural network classifier uses all training examples to learn a hypothesis (model). For practical suitability, the authors focused on a micro cold forming scenario, where a micro cold forming machine produces micro cups. Evaluation with different parameters showed good defect detection results.

In Reference [9], authors presented an application of deep convolutional neural networks (DCNNs) for automatic detection of rail surface defects. For classification, a DCNN was used, based on the classical convolutional neural network proposed by LeCun et al. [10]. The authors developed three models of convolutional networks, which contained 2–3 convolutional layers and 2–3 fully connected layers with different numbers of neurons. After each convolutional layer, max-pooling was used. Samples were classified into three classes. The first class represented the normal rail. The second class contained all types of small defects, and finally the third class only consisted of rail joints. With the proposed DCNNs, the rail defect classes can be successfully classified with almost 92% accuracy. 

Malekzadeh et al. [11] proposed an automatic-image-based aircraft defect detection method using Deep Neural Networks. Dataset images were taken in a straight view of the airplane fuselage. For each image, a binary mask was created by an experienced inspector to represent defects. Each image was partitioned into the 65 × 65 patches with respect to image resolution to include the smallest defect within a single patch. There were two classes of patches: defect and no-defect. Authors have used a convolutional neural network pre-trained on ImageNet as a feature extractor: AlexNet [12] and VGG-F [13] networks. The proposed algorithm achieves about 96.37% accuracy. The proposed algorithm is able to detect almost all the defects of the aircraft fuselage and significantly reduce the workload of manual inspection.

An algorithm for surface defect detection of steel plates was proposed by Tian and Xu [14]. For this purpose, authors used extreme learning machine (ELM)—a fast machine learning algorithm, implemented by a hidden matrix generated with random initialization parameters. The ELM algorithm was proposed by Huang et al. [15] as a kind of single-hidden-layer feedforward neural network. The accuracy of defect detection on testing dataset is 90–94%. CPU time needed for analyzing one image is from 0.03 to 250 s.

Cha et al. [16] proposed a vision-based method using a deep architecture of convolutional neural networks for detecting concrete cracks without calculating the defect features. Analyzed images were cropped into small images with 256 × 256 pixel resolutions for training and validation. The small images were used as the dataset to train the CNN. The test images were scanned by the trained CNN using a sliding window technique, which facilitated the scanning of any images with resolutions higher than 256 × 256 pixel, and the crack maps were consequently obtained. Subsequently, the authors expanded their methodology by offering the Faster-R-CNN-based structural visual inspection method [17]. The method allows detecting five types in the five types of surface damages: concrete cracks, steel corrosion (medium and high levels), bolt corrosion, and steel delamination. Authors showed that Faster R-CNN has better computation efficiency based on the optimized architecture of the network, and it provides more flexible sizes of the bounding boxes to accommodate for different sizes and scales of the input images. In addition, a video-based damage detection framework using the Faster R-CNN was developed, which can provide quasi real-time, autonomous vision-based structural damage detection.

A separate line of research on images is the fractographic analysis, the main tools of which are physics of a solid body (the theory of dislocations, etc.), material science (in the case of building correlations between fracture parameters and the size of grains, inclusions, etc.), optical–digital methods, etc. [18,19].

Until recently, fracture surface parameters of materials and structures were measured, either manually or automatically, but the measurement software was adjusted by the operator. This brought a significant subjectivity and complexity to measurements. Therefore, the need to develop and test new approaches to the automated fractographic analysis of material fractures is very important [20,21].

The peculiarity of working with fractographic images is that, for their analysis, it is necessary not only to identify the area, in which the objects are located, but also to evaluate the morphological features of the objects. The calculation of quantitative parameters of dimples of viscous detachment (their count, size, shape, etc.) will not only allow establishing the causes of material fracture, but will be informative input parameters for models aimed at optimizing their properties. In addition, with a view to fractodiagnostics and the establishment of the causes that lead to fracture of load-bearing structures (especially in aviation and space technology, and nuclear energy), algorithms are needed that make it possible to identify, recognize and calculate the parameters of dimples of detachment, which will provide for their further statistical analysis and high accuracy and repeatability of the experiment.

We should note that in the context of fractographic analysis of the surface of tearing, the disadvantage of the previously considered approaches is either their narrow focus on the solution of a particular task (classification of images, etc.) [8,9,10,11,12,13,14,15], or a zonal result in the form of bounding boxes, which makes it impossible to calculate the morphological parameters of the objects found [16,17]. Therefore, the task of developing a methodology that would enable effective automation of the process of fractographic research remains relevant.

In this paper, we proposed a method for detecting dimples of viscous detachment on fractograms of titanium alloys, which was based on the use of a DCNN. For this purpose, a number of models of neural networks with various sets of hyperparameters have been developed and investigated. Their accuracy and speed were investigated, and the optimal neural network model was selected. The proposed network contained two convolution layers, two subsampling layers, and two fully connected layers. The neural network classified the pixels into two categories: “dimples” and “edges”, and admitted the affiliation of each pixel to one of these classes. To study the neural network, a known algorithm for analyzing fractograms was used. A series of images of the rupture surface of a titanium alloy was analyzed using the proposed neural network. The result obtained was compared to the basic method.

## 2. Methods

To identify dimples of viscous detachment, a convolutional neural network was used for fractograms of the rupture surface of the titanium alloy VT23. All pixels of the image were divided into two classes: these that belong to the dimples of detachment, and those that do not belong to them. To study the neural network, the result of detecting dimples by the previously developed algorithm [22] was used. The essence of this algorithm is in finding the edges of dimples of detachment. It contains the operations of filtering with a set of filters for identify edges, adaptive thresholding, skeletonization, dilation and segregation of connected areas. Each of these steps needs to be customized in practice. Using a trained neural network allows reducing the number of parameters that need to be adjusted manually, which simplifies the process of image analysis.

### 2.1. Training DataSet of the Neural Network

**Figure 1:**
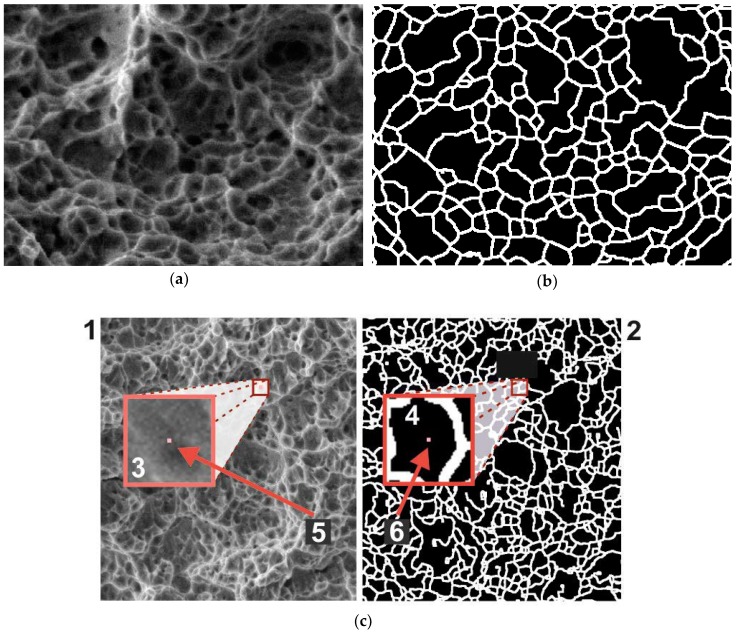
(**a**) Dimples of viscous detachment on the surface of a specimen from titanium alloy VT23; (**b**) the result of their detection using the algorithm [22]; (**c**) a diagram illustrating the principle of training the neural network: (1) training image, (2) image with correct answer labels, (3) zoomed fragment of training image with the size $2w_{k}+1\times 2w_{k}+1$, which is an element of the training set, (4) fragment of the image with labels, which by its position and size corresponds to (3), (5) the central pixel of (3), and (6) the pixel containing the correct answer label for (3).

Figure 1 shows the image of the rupture surface of the titanium alloy obtained from the electronic scanning microscope REM-106I (JSC «SELMI», Sumy, Ukraine). On the basis of the initial images (Figure 1a) and the results of finding dimples by the algorithm [22] (Figure 1b), a training dataset for the convolutional network was created.

Let the initial greyscale image be described by the function $i_{o}\left(x,y\right)$, and the binary image showing the result be expressed by the function $i_{s}\left(x,y\right)$. For each pixel (x,y) of the initial image, a square window with the size $2w_{k}+1\times 2w_{k}+1$ and the coordinates of the angles $\left(x-w_{k},\;y-w_{k}\right),\;\left(x+w_{k},\;y-w_{k}\right)$, $\left(x-w_{k},\;y+w_{k}\right),\;\left(x+w_{k},\;y+w_{k}\right)$ was used. The fragment of the initial image $i_{o}\left(x,y\right)$ that belonged to this window was a single test sample for the neural network (Figure 1c). The label indicating the correct answer for such a fragment was the value of $i_{s}\left(x,y\right)$. Thus, a fragment of the initial image with some central pixels (x,y) was provided at the input sample of the neural network, and the result was obtained at the output, indicating the affiliation of this pixel to one of the two classes: “dimple” (label 0) or “edge” (label 1). To provide a greater variety of training data, the fragments were selected with a step of 3 pixels in both axes. 

Based on 46 fractograms measuring 400 × 277 pixels, a training dataset for the neural network was created, which contained 562,856 image fragments measuring 31 × 31 pixels ($w_{k}=15$). Another 11 fractograms were used for testing. On this basis, a test dataset of 134,596 specimens was generated. Subsequently, the neural networks of different architectures were investigated at different values of $w_{k}$. For $w_{k}$ = 7 and $w_{k}$ = 10, we used a central portion of the desired size (15 × 15 pixels at $w_{k}$ = 7 and 21 × 21 pixels at $w_{k}$ = 10) from the initial fragment size, which was performed as follows. After training the neural network, we used a floating window measuring $2w_{k}+1\times 2w_{k}+1$, which was moved along the fractogram with a step of 1 pixel in both axes of the image. Each section that got into the window was fed on the input of the neural network. Thus, we obtained a prediction for the central pixel of the window whether it belongs to one of the classes.

### 2.2. Architecture of Convolutional Neural Network

**Figure 2:**
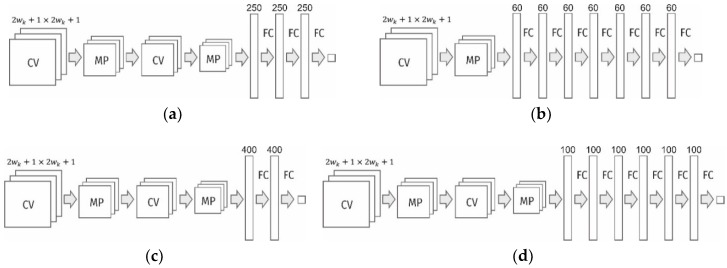
Best by accuracy/speed ratio neural network architectures. (**a**)—neural network with 2 convolutional layers and 3 intermediate fully connected layers; (**b**)—network with single convolutional layer and the stack of 7 small fully connected layers; (**c**)—network with 2 convolutional layers and 2 big fully connected layers (this model showed the best performance); (**d**)—model with 2 convolutional layers and the stack of 6 intermediate fully connected layers; CV—convolutional layers; MP—max-pooling layers; FC—fully connected layers.

Seventeen models of convolutional neural networks were designed and investigated to detect dimples of viscous detachment on surface images. The best neural network architectures are shown in Figure 2. A complete list of the investigated networks is given in Table 1. The neural networks were implemented using the Keras library on the basis of Theano tools [23] and the CUDA Toolkit [24]. CUDA is a parallel computing platform and programming model developed by NVIDIA for general computing on graphical processing units (GPUs). For training and testing of the neural networks, we used a normal desktop PC with GPU NVIDIA GeForce GT 630 M.

In general terms, the structure of these networks contains two blocks of layers: (a) convolutional layers (in pairs with max-pooling layers) to detect spatial features, and (b) fully connected layers to generalize the found features. The input layer in all models consisted of $2w_{k}+1\times 2w_{k}+1$ neurons, and the output layer was composed of two neurons with the activation function Softmax. The first and second output neurons show the probabilities that the central pixel of the image fragment fed at the input belong to the classes “dimple” and “edge”, respectively. For the neurons of the hidden convolutional and fully connected layers, the linear activation function Rectified Linear Unit (ReLU) was used. It can be defined as f(x)=max(0,x). In the previous analysis of the images, the accuracy of the neural network with activation functions such as Sigmoid, Tanh and ReLU was investigated. It was found that the best accuracy was provided by the activation function ReLU, and therefore, it was used in further research.

As loss, the function used categorical cross-entropy. If our model predicts q(x) while the target is P(x), then the categorical cross-entropy is defined as follows: $H\left(P,q\right)=-\sum_{x}P\left(x\right)\log\left(q\left(x\right)\right)$. The cross-entropy measures the performance of a classification model, of which output is a probability value between 0 and 1. As metrics, we use accuracy: the proportion of correct predictions with respect to the targets. For training, CNNs used the optimizer Adam [25], which implements an algorithm for first-order gradient-based optimization of stochastic objective functions, based on adaptive estimates of lower-order moments.

The investigated models of neural networks differed in the number and size of convolutional and fully connected layers, as well as in the size of the input layer. The training process was stopped if, after 15 epochs, the accuracy of the validation data did not exceed the maximum accuracy attained during the previous period.

### 2.3. Comparison of the Developed Neural Networks

The main criteria for choosing the optimal neural network architecture were: the recognition accuracy of the test data and the time of analysis. It was found that for the investigated type of images, the use of more than two convolutional layers does not lead to a significant increase in accuracy, but significantly increases both the training time and the analysis time. Moreover, neural networks with a single convolutional layer and a sufficient number of feature maps are also quite efficient. However, in this case, deep block of fully connected layers is required. A need for a small number of convolutional layers suggests that the features allocated for diagnosis by the neural network are simple. It was also found that it is more efficient to use a bigger number of fully connected layers with a small number of neurons than one fully connected layer with a large number of neurons. This reduces not only the number of training parameters of the neural network, but also the time of its work. The accuracy of the diagnosis remains high.

**Figure 3:**
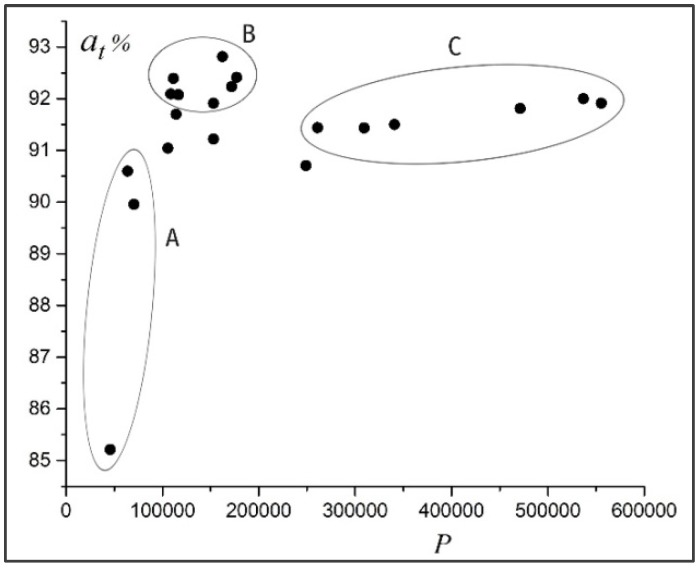
Relationship between the accuracy $a_{t}$ and the number of parameters *P* of the neural network.

Figure 3 shows the averaged accuracy $a_{t}$ of detecting dimples in test images for the investigated neural networks with a different number of training parameters P. The results obtained show that a bigger number of neurons is not a guarantee of high accuracy of the neural network. In Figure 3, we can distinguish three groups of neural networks: *A*—simple, with a small number of parameters and lower accuracy; *B*—more complex, with more parameters and higher accuracy; *C*—the most complex, with the biggest number of parameters and highest accuracy. Most valuable from a practical point of view are neural networks of group *B*, which combine simplicity of structure and accuracy of diagnostics.

At the input of the neural network, fragments of size $2w_{k}+1\times 2w_{k}+1$ pixels were fed. The networks were investigated at $w_{k}=7$, $w_{k}=10$ and $w_{k}=15$. It was found that for the analyzed images, $w_{k}=10$ is optimal. With a small window ($w_{k}=7$), the accuracy of the diagnosis is low. The large window ($w_{k}=15$) allows getting higher accuracy, but its increment is very small compared to the middle window ($w_{k}=10$).

**Figure 4:**
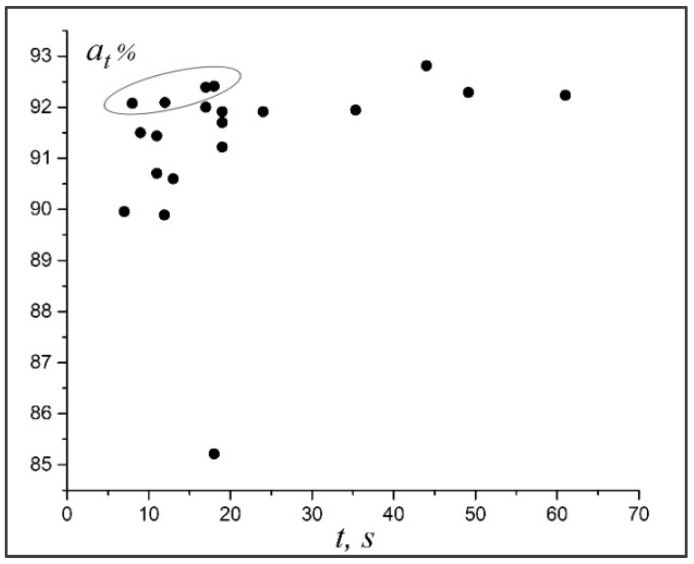
Relatioship between the accuracy $a_{t}$ and the duration of image analysis *t*. The neural networks shown in Figure 2 are highlighted.

Figure 4 illustrates the relationship between the accuracy and time of diagnostics of the neural networks. Of all the architectures, we have four, which combine good performance and accuracy of diagnostics. Their structures are shown in Figure 2. For further practical use and analysis of the dimples of detachment of the titanium alloy according to the fractograms, the neural network with architecture shown in Figure 2c (also Table 1, network #5) was chosen. On average, it ensured the diagnostic accuracy of 92.11% for the test data. The network was trained during 77 epochs. For this network ($w_{k}=10$), the number of training parameters was 108,622, and the time of diagnostics of the image with a size of 400 × 277 pixels was 12 s (on the above equipment).

The processing time of an image can be reduced if we apply a step larger than 1 pixel to a floating window in the analysis of the fractogram. In this case, interpolation of missed values should be performed after the analysis. However, at the same time, the accuracy of detection of the boundary elements of objects will be lost. Since the proposed neural network is used for laboratory analysis of test specimens, the selected processing time was considered acceptable.

**Figure 5:**
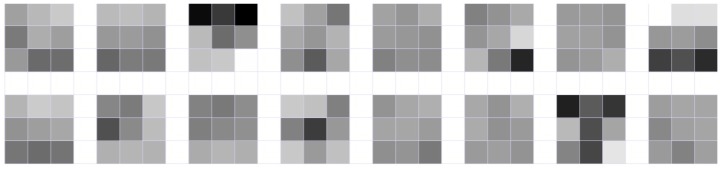
Vizualization of the kernel weights of the trained model. Black cells represent the minimum weight value (−1.262), and white cells represent the maximum weight value (1.010).

The visualization of the weights of 20 kernels of the trained model (Figure 5) shows that the best response will be provided by the above convolutional layer for the pixels that correspond to the edges of the dimples. Here, both the kernels that correspond to vertical/horizontal brightness gradient, and the kernels with a diagonal gradient were presented.

## 3. Results and Discussion

**Figure 6:**
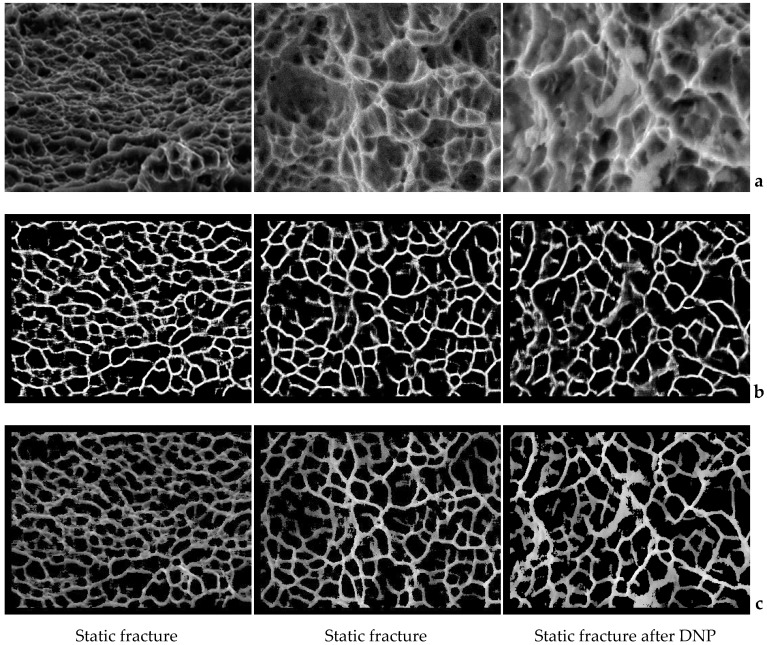
(**a**) SEM images of the titanium alloy VT23M (first image) and VT23 (second and third images) [26]; (**b**) visual representation of the result from the output of the neural network; (**c**) the result of detecting dimples (black spots), which is imposed on the initial SEM images.

Figure 6a shows the fractograms from the test dataset and Figure 6b presents the diagnostic result using the selected neural network model. Fractograms were obtained by an electron microscopy of the rupture surface of titanium alloys VT23 and VT23M. Although during the training of the network, only two labels were used: 0 (affiliation to the dimple) and 1 (affiliation to the edge), a value within the range (0–1) was obtained at the output for each pixel, which indicates the probability belonging to the some class according to the selected neural network model. Let an array of output values obtained for all pixels of image for class “edge” be denoted by $P_{\mathrm{cnn}}\left(x,y\right)$. Visualization of this array is shown in Figure 6b.

**Figure 7:**
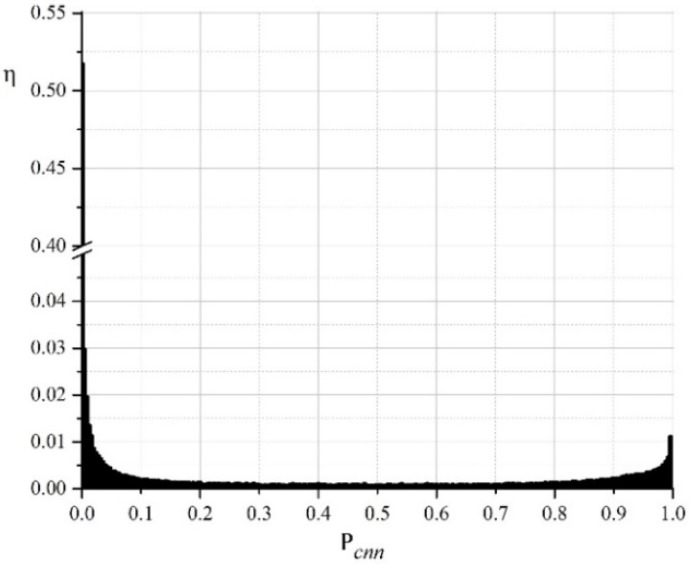
Normalized histogram of distribution of values $P_{\mathrm{cnn}}\left(x,y\right)$. η is the share of image pixels, which corresponds to the value $P_{\mathrm{cnn}}.$

To make an explicit decision on the affiliation of pixels to one of the classes, a specific threshold T needs to be selected. Pixels, for which $i_{\mathrm{cnn}}\left(x,y\right)\leq T$, is considered to belong to the “dimple” class, otherwise to the “edge” class. A typical normalized histogram of the distribution of values $P_{\mathrm{cnn}}\left(x,y\right)$ for one of the investigated images is given in Figure 7. The values $P_{\mathrm{cnn}}$ close to 0 correspond to pixels that are highly likely to belong to the “dimple” class. Values close to 1 correspond to pixels that are highly likely to belong to the “edge” class. As is seen from the histogram, two peaks are grouped around two opposite values—0 and 1. This is a sign of a successful operation of the neural network. The first (higher) peak for $P_{\mathrm{cnn}}\approx 0,\;\ldots ,\;0.15$ corresponds to the “dimple” class, and the second (smaller) peak for $P_{\mathrm{cnn}}\approx 0.85,\;\ldots ,\;1$ corresponds to the “edge” class. In total, these two zones belong to more than 80% of all pixels. The remaining 20% of the pixels are distributed in the range $P_{\mathrm{cnn}}\approx 0.15,\;\ldots ,\;0.85$. Thus, the choice of the boundary T in this range has a negligible effect on the result.

**Figure 8:**
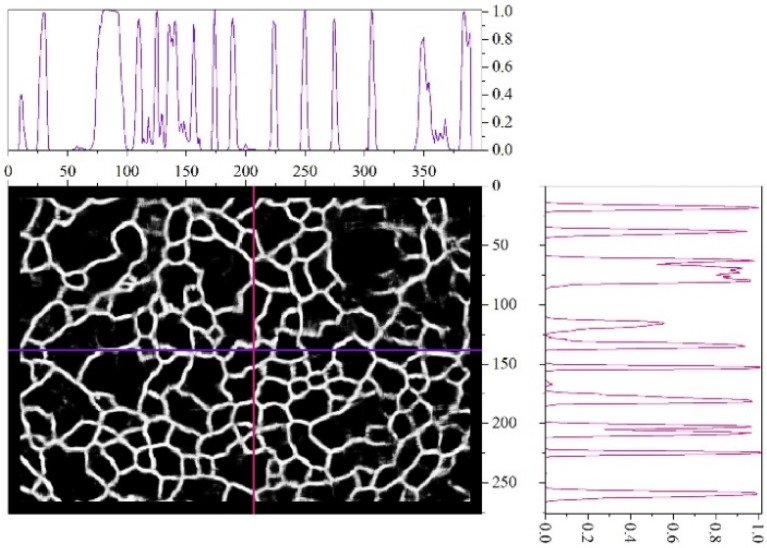
Visualization of the array $P_{\mathrm{cnn}}\left(x,y\right)$ and its cross-sections in two mutually perpendicular directions.

Figure 8 shows the visual representation of the array $P_{\mathrm{cnn}}\left(x,y\right)$ for one of the images and gives its cross-sections along two coordinate axes. The cross-sections show that the peaks corresponding to the edges of the dimples recognized by neural network have a high slope. This confirms the conclusion that choosing the boundary from the middle range of $P_{\mathrm{cnn}}$ has a slight effect on the final result. It is also evident that the heights of these peaks are different in the sections, so the choice of T in the upper range causes loss of some edges. At the same time, even at very low values, i.e., $P_{\mathrm{cnn}}<0.1$, individual dimples are highlighted, since the line slop in the section is high.

Thus, the choice of the boundary value T in the upper range of values ($P_{\mathrm{cnn}}\geq 0.85$; see Figure 7) results in the allocation of pixels that are very likely to belong to the edges, but not all edges can be detected. This can lead to the “coalescence” of adjacent objects—the dimples. Choosing T in the lower range ($P_{\mathrm{cnn}}\leq 0.15$) will provide for the allocation of pixels that are very likely to belong to the dimples. Since the aim of this research is to study the dimples, the boundary value T was chosen in this lower range. For the studied group of images, T=0.1 was used. This corresponds to the pixel allocation to the “dimple” class, for which the neural network has calculated a probability of more than 90%.

Figure 6c shows the results of detecting dimples superimposed on the original images (in order to increase the contrast, we increased brightness in the initial images). The trained neural network clearly identifies the image areas that correspond to the dimples. Interestingly, the visual expert analysis of the results obtained has revealed that the neural network often allocates dimples better than the original method. The most probable explanation for this phenomenon is that the neural network of the selected architecture allocates 40 feature maps, while the original method [22] uses only two spatial filters (therefore, it focuses on two features only).

### Comparison of the Results Obtained Using the Proposed Neural Network with the Results Obtained Using the Initial Algorithm

The developed neural network performs pixel-by-pixel processing of the image, making predictions for each pixel concerning its affiliation to one of the classes. However, the practical value is the calculation of the dimple parameters in the image: their distinct and total area, equivalent diameter, angle of inclination, etc. A comparison of the results obtained on the basis of the proposed neural network with the results obtained by the initial algorithm [22,27,28] shows their high similarity (Table 2).

**Figure 9:**
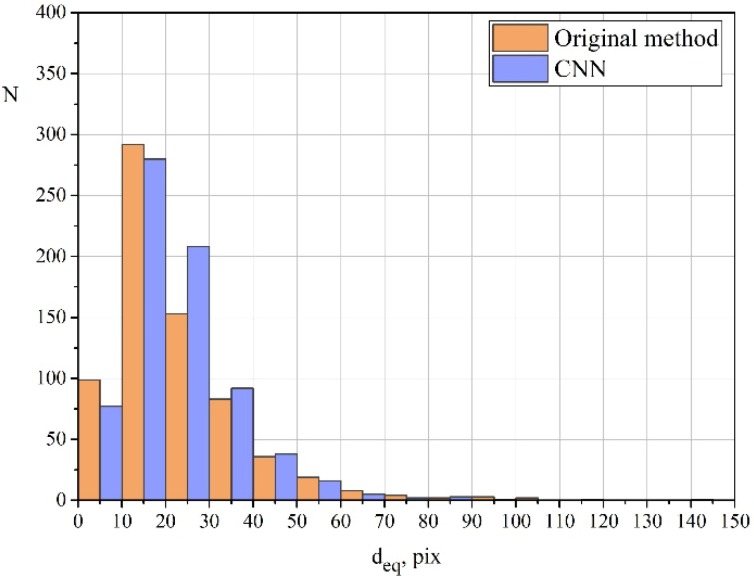
Diagram of the distribution of equivalent diameters, calculated using the original method [22] and the proposed CNN.

Figure 9 shows a distribution diagram of equivalent diameters for one of the investigated images calculated using both methods. The diagram illustrates several features of the proposed approach compared to those of the original method:

- The neural network detects a smaller amount of small dimples. This can be explained by the fact that the old method is focused on finding brightness differences; therefore, dimples are sometimes erroneously detected on homogeneous parts of the image;
- The neural network also detects a smaller amount of large dimples. This is due to the greater sensitivity of the neural network, which takes into account significantly more feature maps. As a result, large dimples become divided into several smaller ones;
- The neural network detects a bigger amount of middle-sized dimples. This amount is likely to increase due to the division of some large dimples and the coalescence of some small dimples.

## 4. Conclusions

In this paper, we proposed a method for detecting dimples of viscous detachment on fractographic images using a 4-layer convolutional neural network, which allocates each pixel of the input image to one of two classes: “dimple” or “edge”. The neural network was trained on the basis of the results obtained using the previously developed algorithm. The neural network was tested on surface images of titanium alloys VT23 and VT23M. Experimental results show high accuracy of the method and indicate the possibility of its practical application. It is revealed that in the general case, the neural network recognizes the sought objects better than the original method based on the set of filters. Based on the results obtained with the model, dimple parameters such as their area, number, equivalent diameter, shape factor, visual depth, inclination, etc., are calculated. Having these parameters for the whole set of surface dimples, it is possible to perform their statistical analysis and make conclusions about the properties of the material.

## Figures and Tables

**Table 1:** Structure and characteristics of the investigated neural networks. CV—convolutional layers (the brackets indicate the number of feature maps and the size of the kernels), MP—max-pooling layers (the brackets show the size of the pooling window), FC—fully connected layers (the brackets indicate the number of neurons).

No	CNN Model Description	w_k_, px	Average Test Accuracy, %	Diagnostic Time, s
1.	CV(32 × 3 × 3)–MP(2 × 2)–CV(32 × 3 × 3)–MP(2 × 2)–CV(32 × 3 × 3)–CV(32 × 3 × 3)–FC(1024)	15	92.81	45
2.	CV(32 × 3 × 3)–MP(2 × 2)–CV(32 × 3 × 3)–MP(2 × 2)–CV(32 × 3 × 3)–FC(250)–FC(250)–FC(250)	10	92.41	19
3.	CV(32 × 3 × 3)–MP(2 × 2)–CV(32 × 3 × 3)–MP(2 × 2)–FC(100)–FC(100)–FC(100)–FC(100)–FC(100)–FC(100)	10	92.39	17
4.	CV(32 × 5 × 5)–MP(2 × 2)–CV(64 × 3 × 3)–MP(2 × 2)–CV(32 × 3 × 3)–FC(1024)	15	92.23	61
5.	CV(32 × 3 × 3)–MP(2 × 2)–CV(32 × 3 × 3)–MP(2 × 2)–FC(400)–FC(400)	10	92.11	12
6.	CV(32 × 3 × 3)–MP(2 × 2)–FC(60)–FC(60)–FC(60)–FC(60)–FC(60)–FC(60)–FC(60)	10	92.08	8
7.	CV(32 × 3 × 3)–MP(2 × 2)–CV(32 × 3 × 3)–MP(2 × 2)–FC(1024)	10	92.00	17
8.	CV(32 × 3 × 3)–MP(2 × 2)–CV(32 × 3 × 3)–CV(32 × 3 × 3)–CV(32 × 3 × 3)–FC(1024)	10	91.91	24
9.	CV(32 × 3 × 3)–MP(2 × 2)–CV(32 × 3 × 3)–MP(2 × 2)–CV(32 × 3 × 3)–FC(1024)	10	91.91	19
10.	CV(32 × 3 × 3)–MP(2 × 2)–CV(32 × 3 × 3)–MP(2 × 2)–CV(32 ×3 × 3)–FC(200)–FC(200)	10	91.70	19
11.	CV(32 × 3 × 3)–MP(2 × 2)–FC(100)–FC(100)–FC(100)	10	91.50	9
12.	CV(48 × 3 × 3)–MP(2 × 2)–FC(50)–FC(50)–FC(50)–FC(50)–FC(50)–FC(50)–FC(50)–FC(50)–FC(50)	10	91,44	11
13.	CV(32 × 3 × 3)–MP(2 × 2)–CV(32 × 3 × 3)–MP(2 × 2)–CV(32 × 3 × 3)–FC(1024)	10	91.22	19
14.	CV(32 × 3 × 3)–MP(2 × 2)–CV(32 × 3 × 3)–CV(20 × 3 × 3)–FC(400)–FC(400)	7	90.70	11
15.	CV(32 × 3 × 3)–MP(2 × 2)–CV(32 × 3 × 3)–CV(32 × 3 × 3)–CV(32 × 3 × 3)–FC(1024)	7	90.60	13
16.	CV(20 × 3 × 3)–MP(2 × 2)–CV(30 × 3 × 3)–MP(2 × 2)–FC(200)–FC(200)	7	89.95	7
17.	CV(20 × 5 × 5)–MP(4 × 4)–CV(32 × 3 × 3)–MP(4 × 4)–FC(1024)	15	85.21	18

**Table 2:** Comparison of the results obtained using the neural network and method [22].

Parameter	Deviation of the Result of the Developed Neural Network in Relation to the Result of Method [22]
The total area of the dimples	<4.0%
The number of dimples	<4.0%
Average equivalent diameter of the dimple	<3.0%
